# UVA/UVA1 phototherapy and PUVA photochemotherapy in connective tissue diseases and related disorders: a research based review

**DOI:** 10.1186/1471-5945-4-11

**Published:** 2004-09-20

**Authors:** Frank Breuckmann, Thilo Gambichler, Peter Altmeyer, Alexander Kreuter

**Affiliations:** 1 Department of Dermatology, Ruhr-University Bochum, 44791 Bochum, Germany; 2 Dermatology Out-Patient Clinic, Oldchurch Hospital, Romford RM7 OBE, Greater London, UK

## Abstract

**Background:**

Broad-band UVA, long-wave UVA1 and PUVA treatment have been described as an alternative/adjunct therapeutic option in a number of inflammatory and malignant skin diseases. Nevertheless, controlled studies investigating the efficacy of UVA irradiation in connective tissue diseases and related disorders are rare.

**Methods:**

Searching the PubMed database the current article systematically reviews established and innovative therapeutic approaches of broad-band UVA irradiation, UVA1 phototherapy and PUVA photochemotherapy in a variety of different connective tissue disorders.

**Results:**

Potential pathways include immunomodulation of inflammation, induction of collagenases and initiation of apoptosis. Even though holding the risk of carcinogenesis, photoaging or UV-induced exacerbation, UVA phototherapy seems to exhibit a tolerable risk/benefit ratio at least in systemic sclerosis, localized scleroderma, extragenital lichen sclerosus et atrophicus, sclerodermoid graft-versus-host disease, lupus erythematosus and a number of sclerotic rarities.

**Conclusions:**

Based on the data retrieved from the literature, therapeutic UVA exposure seems to be effective in connective tissue diseases and related disorders. However, more controlled investigations are needed in order to establish a clear-cut catalogue of indications.

## Background

Unlike UVB radiation that can penetrate at the most into the papillary dermis, longer wavelengths in the UVA region have the capacity to reach the subcutis as well. Accordingly, as well as due to its lesser antiproliferative activity, UVB irradiation has not been established in the treatment of sclerotic disorders except for occasional cases of graft-versus-host disease (GvHD) [1,2]. Hence, this review examines different modalities of UVA phototherapy in the treatment of connective tissue diseases and related disorders.

The term irradiance (e.g., in mW/cm^2^), which is the most commonly used term in photobiology, relates to the subject (e.g., patient) struck by the irradiation. In photobiology, the time integral of the irradiance is commonly expressed as fluence (e.g., in J/cm^2^), or even more loosely as dose [3,4]. Even though mostly combined with 8-methoxypsoralene or other photochemotherapeutic agents, broad-band UVA irradiation (315–400 nm), containing both UVA1 (340–400 nm) and UVA2 (315–340 nm), was used as monotherapy e.g. in the treatment of atopic dermatitis [5]. However, this phototherapeutic option was being replaced more frequently by the usage of irradiation devices which allow a more effective treatment by the administration of selected spectra. By eliminating shorter wavelengths in the UVA2 region adverse effects such as erythema are minimized and therapeutically effective higher UV doses can be administered. Thus, today broad-band UVA may play a subordinated role in modern phototherapy anymore, except for the combined application with psoralenes, even though it is still used for a large number of patients due to its wide availability and although it has not yet been directly compared with UVA1 for many sclerosing disorders. Conventional UVA1 treatment emitts wavelengths mainly between 340 and 400 nm, but may also produce scattered radiation >530 nm including infrared irradiation (780–3000 nm). Three different pattern of UVA1 dosage have been described: high-dose UVA1 phototherapy applying UVA1 doses ranging from 90–130 J/cm^2 ^single (975–1840 J/cm^2 ^cumulative) irradiation, medium-dose UVA1 phototherapy admitting doses between 20–90 J/cm^2 ^single (300–975 J/cm^2 ^cumulative) UVA1 and last but not least low-dose UVA1 phototherapy administering single UVA1 doses of ≤ 20 J/cm^2 ^or a cumulative doage ≤ 300 J/cm^2^, respectively [6-9]. Conventional UVA1 phototherapy may be accompanied by extensive heat load predominantly generated by infrared irradiation (780–3000 nm) and/or insufficient cooling systems of the phototherapy devices. For this reason, within the last years, lavish new UVA1 equipment was developed containing a special filtering and cooling system, in which a considerable amount of wavelengths >530 nm are eliminated and consequently the heat load due to heat-producing infrared radiation is strongly diminished [10]. Since more than two decades, the combination of oral 5- or 8-methoxypsoralen followed by broad-band UVA exposure is an effective treatment option in a widespread number of indications such as psoriasis and cutaneous T cell lymphoma [11-13]. Psoralens specifically belong to the best characterized agents of photosensitizing chemicals. Following its administration low-dose UVA irradiation is applied including an individual progression depending on the formation of the so-called PUVA erythema. In general, the overall mean cumulative dose has been found to comprise ≤ 400 J/cm^2 ^UVA [14]. In contrast to systemic PUVA therapy topical PUVA, for example applied as PUVA bath, PUVA shower or PUVA cream, is mainly characterized by absent/reduced systemic side effects and the restriction to a selective limited lesional area [15]. As a consequence, systemic PUVA has mostly been replaced by topical PUVA treatment representing an efficient well-tolerable alternative to oral methoxypsoralen administration. Generally, overall mean cumulative dosage has been found to comprise ≤ 200 J/cm^2 ^UVA [14].

Different types of UVA phototherapy were introduced as an innovative and promising therapeutic option in the treatment of inflammatory diseases such as atopic dermatitis and more recently in therapy of lymphoproliferative disorders such as cutaneous T cell lymphoma and related skin affections. No more than seven years ago, subsequent to promising clinical results of extracorporeal photochemotherapy and psoralene plus UVA (PUVA) in systemic sclerosis (SSc) and morphea [16-19], first investigations have verified the therapeutic value of UVA1 irradiation for the treatment of localized scleroderma (LS) [20-22].

However, encouraged by the clinical success and the diversity of immunomodulatory effects achieved by the use of UVA phototherapeutic regimens in a great number of different indications, additional studies focused on investigating the efficacy of UVA phototherapy in the treatment of a widespread range of sclerotic skin diseases [23]. Furthermore, series of UVA exposure may be used in the treatment of lupus erythematosus (LE), even if known as a photosensitive condition.

Today, different forms of UVA phototherapy are widely used and have subsequently developed into a treatment modality of importance within the field of dermatology and rheumatology at least as an adjunctive treatment and, beside the 'initial indications', may also or even especially be indicated as a successful alternative in the treatment of skin manifestations of connective tissue diseases and related disorders.

## Methods

For this systematic review we concentrated on the therapeutic use of UVA application in humans. The computerized bibliographic database PubMed (includes all citations from Medline and additional life science journals) without time limits (January 1966 to April 2004) was screened for original papers, case reports, letters, reviews and book articles on UVA/UVA1/PUVA. As main key words we used "UVA", "UV-A", "UVA phototherapy", "UVA1", "UV-A1" "UVA1 phototherapy", "PUVA", "PUVA phototherapy", "PUVA photochemotherapy", "ultraviolet A", "ultraviolet A1", "sclerosis", "scleroderma", "sclerosus", "sclerotic", "sclerodermoid", "morphea", and "lupus erythematosus". Other sources included monographs, textbooks, and the reference lists from all the articles retrieved. All abstracts were read and selected by two authors (F.B., T.G.) Inclusion or exclusion of articles were based on consensus. Relevant data including study design, number of patients, duration of treatment, clinical outcome, cumulative UVA doses, and adverse effects were retrieved from the articles, summarized and briefly discussed as follows.

## Results

### Systemic sclerosis

SSc, affecting the connective tissue of various organs including the skin, is histologically recognized by an alteration of the microvasculature with a rarefaction of the vessels within the papillary layer, perivascular skin infiltrating T lymphocytes (mainly due to an alteration of the Th2 immune response), rapid proliferation of dermal fibroblasts exhibiting an elevated status of protein synthesis and by a resulting massive deposition of collagen in both the skin and internal organs [24-27].

#### UVA1

So far UVA1 phototherapy has only been reported to be effective in the clearance of acral sclerotic skin lesions of SSc patients by administering local UVA1 irradiation of the hands or forearms, respectively. First, Kobyletzki *et al. *reported on preliminary results about the efficacy of low-dose UVA1 phototherapy within the treatment of acrosclerosis in eight patients suffering from progressive SSc [28]. Exposure of 30 J/cm^2 ^UVA1 was administered four times per week for eight weeks and subsequent three times per week for six weeks resulting in a total of 50 sessions and a cumulative dose of 1500 J/cm^2^. In 2000, Morita *et al. *could confirm the UVA1-induced softening of sclerosis following partial body 60 J/cm^2 ^medium-dose UVA1 phototherapy ranging from 510 to 1740 J/cm^2 ^cumulative dose in three patients with diffuse and one with limited SSc, later further underlined by decreased dermal decorin levels as published in 2003 [29,30]. A recent open non-randomized study including 18 patients with SSc derived acrosclerosis revealed softening of former stiffness, an increase of total skin distension, the reduction of skin thickness and an elevation of dermal collagenase activity in 16 patients following the corresponding irradiation protocol as described by Kobyletzki *et al. *[31]. However, whole-body UVA1 phototherapy has not yet been described, although a possible systemic impact due to the deep penetration depth seems to be imminent.

#### PUVA

Based on first studies reporting the efficacy of topical PUVA in one patient with SSc [7], Kanekura et al. described the positive outcome of former sclerotic lesions in three patients exhibiting cutaneous manifestation of progressive SSc [32]. PUVA was administered for three to eight weeks with daily doses of 0.25 J/cm^2 ^to 0.4 J/cm^2 ^(cumulative dosage: 3.5 J/cm^2 ^to 9.6 J/cm^2^) resulting in remarkable clinical improvement of skin sclerosis index as well as stiffed fingers, hands and knees. Another small uncontrolled study investigated oral PUVA therapy for SSc. The study included four women suffering from SSc receiving a PUVA protocol consisting of 0.5 to 4 J/cm^2 ^single dose UVA given three times a week for ten weeks and a mean cumulative dose of 70.5 J/cm^2 ^UVA. Even though posttherapeutic skin severity scores did not alter significantly, at least microscopic analysis of the histological skin scores of all patients revealed visible improvements [33]. The last case report appeared in 2003 discussing the use of PUVA bath in a young girl, proposing that PUVA bath could also be used in childhood [34].

### Localized scleroderma

LS is characterized by circumscribed fibrotic plaques generally affecting the whole dermis. Thus LS, particularly when occurring in childhood, may contribute to progressive and long-lasting induration of the skin and subcutaneous tissue, growth retardation, muscle atrophy and, in severe cases, even to flexion deformities and poorly healing ulcerations [35].

#### UVA

As to our knowledge, there has been conducted only two large study investigating low-dose broad-band UVA exposure in morphea [36,37]. Twelve patients were irradiated by doses of 20 J/cm^2 ^broad-band UVA three times a week for a total of 20 sessions (400 J/cm^2 ^cumulative dose). Following treatment, all patients experienced marked softening of former skin lesions accompanied by significant reduction of the mean concentration of collagen. Additionally, nine patients received 10 J/cm^2 ^UVA single and 200 J/cm^2 ^UVA cumulative dose. Even though different study parameters varied between both groups, no statistically significant differences could be detected in the clinical response to those doses.

#### UVA1

High-dose UVA1 phototherapy of LS has been introduced by Stege *et al. *in 1997 [38]. Ten patients receiving 130 J/cm^2 ^high-dose UVA1 therapy (30 sessions, 3900 J/cm^2 ^UVA1 cumulative dose) were compared with seven patients treated by low-dose UVA1 phototherapy (30 sessions, 600 J/cm^2 ^cumulative dosage) and internal controls. The authors state that high-dose UVA1 significantly reduced skin thickness and stiffness and increased elasticity of plaques. High-dose UVA1 was superior to low-dose UVA1. By contrast, a number of different studies and case series could also confirm the effectiveness of low-dose UVA1 phototherapy. In 1995, Kerscher *et al. *were able to discuss the first successful phototherapeutic approach of low-dose UVA1 phototherapy of LS [39]. Subsequently, the authors conducted a larger study including 20 patients suffering from LS. Patients were irradiated with low-dose UVA1 for twelve weeks (total of 30 treatment sessions, 20 J/cm^2 ^single dose, 600 J/cm^2 ^cumulative dose) resulting in significant clinical improvement in about 80% of the patients [20]. Two patients exhibiting subcutaneous LS did not improve. Finally, Gruss *et al. *analyzed and compared the effect of their low-dose UVA1 irradiation protocol on late-stage lesions, inflammatory lesions and late-stage lesions with overlying lichen sclerosus et atrophicus (LSA) [40]. All three patients responded well to therapy. In addition to low- and high-dose UVA1, medium-dose UVA1 phototherapy stands for a further phototherapeutic option. In 2001, seven patients with morphea were treated by 30 J/cm^2 ^medium-dose UVA1 phototherapy three times weekly during a ten week period [41]. All patients reported improvement as judged by softening of the skin lesions. Recently, controlled medium-dose UVA1 treatment was performed in a total of eight patients using 48 J/cm^2 ^UVA1 [42]. Irradiation was administered four times per week for twelve weeks resulting in an improvement of skin sclerosis by a cumulative dose of 2304 J/cm^2 ^UVA1. Furthermore, combined therapy with calcipotriol ointment and low-dose UVA1 phototherapy seems to be highly effective at least in childhood morphea. Following a first case report, Kreuter *et al. *conducted a large open prospective study including 19 children suffering from LS [43,44]. UVA1 exposure was given four times a week for ten weeks (20 J/cm^2 ^single dose UVA1, cumulative dose: 800 J/cm^2^) as an adjunct to twice daily topical calcipotriol application. Combined therapy resulted in a relative reduction of clinical scores of about 67%. Its successful use has also been reported regarding the variant of LS en coup de sabre (30 J/cm^2 ^UVA1, 30 sessions) [45].

#### PUVA

First application of PUVA bath photochemotherapy in two cases of LS was published in 1994 by Kerscher *et al*. UVA irradiation was administered once daily four times a week for five consecutive weeks followed by twice per week for additional five weeks (30 treatments, maximum single dose of 20 J/cm^2^), leading to an almost clearance of lesional skin [19]. Evaluation of 17 consecutive patients receiving PUVA bath photochemotherapy (0.2 J/cm^2 ^to 0.5 J/cm^2 ^initial dosage, 1.2 J/cm^2 ^to 3.5 J/cm^2 ^UVA maximum dose) revealed marked clinical improvement in 13 of 17 persons even after 15 treatment sessions [46]. As already mentioned above, Kanekura *et al. *could also verify the effectiveness of PUVA therapy, beside three patients with SSc, in one patient exhibiting generalized morphea [32]. Within the following years, a number of case reports and serial cases were able to reproduce the positive results of PUVA therapy in LS, employing higher initial and cumulative doses and more treatment sessions to achieve improvement/clearance [47-49]. Recently, Pasic *et al. *demonstrated that local PUVA bath may also be of certain benefit for LS in childhood [34]. Moreover, PUVA cream therapy has been successfully introduced by Grundmann-Kollmann *et al. *in four LS patients (cumulative dose ranging from 67.5 J/cm^2 ^to 121 J/cm^2^, maximum single dose: 3.5 J/cm^2^) and, analogous to UVA1 phototherapy, improvement of linear scleroderma en coup de sabre treated with topical calcipotriol ointment and PUVA cream could be observed by Gambichler *et al*. [50,51].

### Extragenital lichen sclerosus et atrophicus

Extragenital LSA is an uncommon skin disease characterized by white porcelain-like sclerotic skin lesions predominantly affecting the flexor surface of the wrists, the upper part of the trunk, and the axillae. In the common form of LSA, genital involvement with atrophy of the vulval, penile, and perianal skin is usually observed [52]. Although LSA has sometimes been considered as a subspecies of LS, LSA is generally regarded as a separate entity as to its distinct clinical and histomorphological peculiarities [53-55].

#### UVA1

The efficiency of UVA1 phototherapy in extragenital LSA was first established by Kreuter *et al. *in 2001 [56]. The authors here report on the improvement of skin status following 40 sessions of long-wave UVA1 irradiation (four sessions per week for ten weeks, total of 40 treatments, 20 J/cm^2 ^low-dose UVA1 per session, 800 J/cm^2 ^cumulative dose). In the same year, a subsequent double casuistic was presented, both receiving the same low-dose UVA1 phototherapy and both responding to therapy with an almost complete clearance of formerly sclerosing lesions [57]. Only one year later, Kreuter *et al. *were able to present the improvement of extragenital LSA in ten patients, all being treated by the established standard irradiation protocol [58]. In contrast, low-dose UVA1 phototherapy of morphea with overlying LSA could not completely reverse the corresponding histopathological changes in a clinical trial in one patient [40].

#### PUVA

As far as PUVA therapy in extragenital LSA is concerned, one case report could demonstrate a promising therapeutic attempt [59]. Interestingly, single UVA1 progressed from 0.3 to 2.3 J/cm^2 ^resulting in a cumulative dose of 31.7 J/cm^2 ^during a six week period. In addition to the extragenital manifestation, PUVA cream photochemotherapy has also been proven to be even effective in genitoanal lesions of LSA [60]. Nevertheless, despite the absence of any short-term side effect, UVA irradiation of genital affections should be performed extremely carefully in order to prevent long-term negative adverse consequences.

### Sclerodermoid graft-versus-host disease

Chronic graft-versus-host disease (GvHD) is an immunological condition frequently occurring as a late consequence of allogenic bone marrow transplantation. Two subtypes, cutaneous lichenoid and sclerodermoid, have been described, based on clinical and histopathological examinations. Sclerodermoid GvHD is a severe adverse immunologic reaction with deposition of collagen in the skin and possibly other soft tissues, resulting in loss of range of motion and functional capabilities [61].

#### UVA1

In 2000, Grundmann-Kollmann *et al. *presented a patient with chronic sclerodermic GvHD, who did not respond to conventional chemotherapeutic agents [62]. Low-dose UVA1 phototherapy was successfully administered four times a week over six weeks (20 J/cm^2 ^single dose, 480 J/cm^2 ^cumulative dose) combined with mycophenolate mofetil therapy. Based on the potentially beneficial effect of UVA1 phototherapy in scleroderma, Staender *et al. *investigated the efficacy of low- or medium-dose UVA1 phototherapy, respectively [63]. Five patients (two of them after insufficient PUVA treatment) received 50 J/cm^2 ^single-dose UVA1 irradiation five times per week for two months followed by a subsequent reduction towards three times weekly. One patient was treated by a stable dosage of 20 J/cm^2 ^in combination with immunosuppressives and extracorporeal phototherapy. In all cases, therapy led to softening of formerly stiffed sclerotic lesions. Most recently, the positive effect of UVA1 irradiation was underlined by Calzavara Pinton *et al*. [64]. Five patients exhibiting sclerodermoid GvHD (localized: 4; generalized: 1) were treated with medium-dose UVA1 phototherapy three times weekly (50 J/cm^2 ^single dose, 750 J/cm^2 ^to 1650 J/cm^2 ^UVA1 cumulative dose) resulting in a complete remission in three and a partial improvement in two patients.

#### PUVA

Already years ago it has been shown that systemic PUVA therapy might be of certain benefit to chronic lichenoid and recalcitrant stages of GvHD, but remains insufficient in sclerotic forms [65-67]. In 1991, another report of PUVA therapy for chronic GvHD could only demonstrate clinical improvement in lichenoid lesions, sclerodermoid skin involvement did not respond to therapy [68]. Oncoming studies including up to 40 patients were able to verify the efficiency of therapeutic PUVA administration, even though only single cases of slightly improved sclerodermatous lesions could be observed [69,70]. However, most recently, Leiter *et al. *performed a successful PUVA treatment in two patients suffering from sclerodermoid GvHD [71]. Inasmuch as improvement of skin involvement is concerned, after a median of 25 treatment sessions, PUVA treatment resulted in a sharp reduction of skin thickness reflected by a relative decrease of 72%. Bath PUVA was administered three to four times per week at the beginning followed by a subsequent reduction via twice to finally once weekly until improvement occurred (34 or 25 sessions, respectively; standard protocol not described; 64.0 or 14.2 J/cm^2 ^cumulative UVA1, respectively). One of both received additional 667 J/cm^2 ^UVA1 irradiation. Nevertheless, most authors state that UVA irradiation should only be performed as an adjunct treatment in addition to conventional chemotherapeutic regimens.

### Lupus erythematosus

LE is an autoimmune disease including a wide spectrum of manifestations in various organ systems. LE specific skin lesions can be found in over 80% of the patients. Currently, three epidemiological forms are distinguished: discoid LE, subacute cutaneous LE and systemic LE beside the presence of a variety of specific subtypes [72].

#### UVA1

The first study on UVA1 phototherapy in subacute cutaneous LE appeared in 1993 [73]. A nine week series of UVA1 phototherapy leading to a cumulative dose of 186 J/cm^2 ^had been administered. Thereafter, an impressive improvement of LE lesions was noted. In another uncontrolled study conducted in 1994, ten patients with systemic LE were treated with 6 J/cm^2 ^for 15 sessions during a period of three weeks [74]. Four of them continued treatment for eight months. The authors could verify a marked clinical improvement combined with a decrease of autoantibody concentration. Since then, two randomized double-blind placebo-controlled cross-over studies were performed. First, McGrath *et al. *reported in a two-phase study two groups of patients, one receiving 6 J/cm^2 ^UVA1 five times a week for three weeks followed by a three week exposure of placebo visible light, the other vice versa [75]. Twenty-five patients completed this phase of the study. Both procedures were followed by an unblinded exposure of progressively decreasing UVA1 levels. Taking clinical as well as serological data in account, the authors proposed that low-dose UVA1 phototherapy might be superior to visible light irradiation. Second, Poldermann *et al. *tried to compare exactly the two different groups in a total of eleven patients [76]. Although no statistically significant difference between the two groups could be evaluated after an exposure of three weeks including 6 J/cm^2 ^cold-light UVA1 five times weekly, significant clinical improvement was restricted to the UVA1 group. Apart from the short term benefit following UVA1 phototherapy, Molina *et al. *were also able to describe a long term benefit following low-dose UVA1 treatment (once/twice per week, 6–15 J/cm^2^) for a mean impressive period of 3.4 years in six patients of their former study [77]. Additionally, recent data of a case report suggest that UVA1 might contribute to a reversal of brain dysfunction and may also improve covered discoid lupus lesions via unknown systemic pathways [78]. As to our knowledge, no positive effects of PUVA treatment have been reported so far.

### Sclerotic rarities

Eosinophilic fasciitis is a rare disorder disabling joint motility closely related to profound morphea with a variable response to treatment [79].

#### UVA1

A case report could verify a significant clinical improvement of eosinophilic fasciitis with low dose UVA1 therapy four times a week for ten weeks, resulting in a total of 40 treatment sessions, additional to azathioprine treatment. At each treatment session 20 J/cm^2 ^UVA1 were applied, resulting in a cumulative dose of 800 J/cm^2^. Afterwards the induration had softened markedly and the patient was able to close the fist again [80].

#### PUVA

Eosinophilic fasciitis successfully treated with PUVA bath photochemotherapy was described by Schiener *et al. *in 2000 [81]. In their study the authors present a case report of single dose 0.3 J/cm^2 ^PUVA four times a week for period of 35 treatments including a progression of 0.3 J/cm^2 ^every third session. Subsequently, irradiation frequency was reduced to three times a week for three weeks or two times a week for another two weeks resulting in a total of 50 treatment sessions and a cumulative dose of 102.1 J/cm^2^.

Pansclerotic morphea of childhood represents a severe variant of LS, often lethal even in young patients [82].

#### UVA1

In 1997, Gruss *et al. *reported the successful administration of low-dose UVA1 phototherapy in disabling pansclerotic morphea of childhood by the usage of 20 J/cm^2 ^four times a week for eight weeks resulting in a total of 32 treatment sessions, a cumulative dose of 640 J/cm^2 ^UVA1 and a remarkable softening of the skin [83]. Another recent study could underline the efficacy of UVA exposure [84].

#### PUVA

In 1995, Scharffetter-Kochanek *et al. *presented a successful approach of PUVA therapy in disabling pansclerotic morphea of a young girl [85]. UVA was administered by a maximum singe dose of 1.8 J/cm^2 ^four times weekly for the first two months followed by maintenance on two treatments per week for another six months. In contrast, an additional case report demonstrated one patient failing to respond to PUVA therapy as an adjunct to penicillamine treatment [86].

Scleromyxedema is a variant of lichen myxedematosus exhibiting erythematous, sclerotic and stiffed lesions beside lichenoid papules caused by an extensive dermal deposition of glycosaminoglycans with only little tendency of spontaneous remission [87-89].

#### PUVA

Following a first promising attempt of PUVA treatment as early as 1984 [90], Adachi *et al. *tried systemic PUVA photochemotherapy in lichen myxedematosus administering 35 treatment session at a cumulative dose of 202 J/cm^2 ^[91]. In this respect, the authors speculate on the inhibition of dermal fibroblasts and synthesis of mucopolysaccharides as a possible mechanism of action. Nevertheless, Schirren *et al. *achieved only limited beneficial effect after combined chlorambucil and PUVA therapy [92].

Scleredema adultorum Buschke, occurring secondarily to diabetes or independently, is an uncommon party sclerodermoid disease characterized by erythematous indurated skin and a mucinous dermal infiltration exhibiting increased collagen deposition [93,94].

#### PUVA

Both PUVA bath and PUVA cream have been reported to be of benefit in patients suffering from Buschke's disease. First, bath PUVA therapy was tested in 1998 by Hager *et al. *in case of three patients exhibiting resistant scleredema adultorum [95]. A median of 59 treatments and a cumulative dose of 245.7 J/cm^2 ^UVA was applied resulting in a substantial clinical improvement in all three patients. Later, Grundmann-Kollmann and co-workers introduced cream PUVA in a patient responding excellently to UVA irradiation (35 sessions, 114.5 J/cm^2 ^total cumulative dose) [96]. As to our knowledge, UVA/UVA1 phototherapy have so far not been taken into account neither in scleredema adultorum nor in scleromyxedema.

POEMS syndrome, characterized by polyneuropathy, organomegaly, endocrinopathy and elevated levels of a monoclonal protein, often exhibit scleroderma-like skin changes [97].

#### UVA1

Severe therapy-resistant cutaneous sclerodermatous lesions of one patient suffering from POEMS syndrome showed a remarkable improvement following low-dose UVA1 phototherapy given for 35 treatment sessions [98].

Bleomycin-induced SSc-like scleroderma may occur following application of the antitumor agent bleomycin [99].

#### UVA1

The authors report a case of drug induced scleroderma after bleomycin administration given due to a malignant testicular seminoma. Low-dose UVA1 phototherapy (20 J/cm^2 ^UVA1, three to four times a week) caused an initial improvement of skin condition, but could not stop the overall progress [99].

Pansclerotic porphyria cutanea tarda is an uncommon subtype of cutaneous porphyria [100]. Simultaneously, massive exposure of organic solvents may also result in sclerotic modifications.

#### UVA1

In 2003, Karamfilov *et al. *could stop progression of skin affection by the use of medium-dose UVA1 phototherapy combined with intensive physiotherapy and oral glucocorticoids in a patient with pansclerotic porphyria cutanea tarda after chronic exposure to organic solvents [101]. UVA1 irradiation was applied for a total of 30 sessions, a single-dose of 40 J/cm^2 ^and a corresponding cumulative dose of 1200 J/cm^2^.

## Discussion

Beside a high number of different side indications, systemic and especially topical PUVA treatment have been shown to provoke a remarkable clearance of psoriatic plaques and infiltrated lesions of cutaneous T cell lymphoma. On the other hand, UVA1 phototherapy achieved practical value in the treatment of inflammatory and malignant T cell related skin diseases. Typical indications regularly include exacerbated atopic dermatitis, cutaneous T cell lymphoma, parapsoriasis or mucinosis follicularis due to the induction of T cell apoptosis and dermal immunoregulation. By reason of notable collateral induction of dermal collagenase activity, UVA irradiation was subsequently introduced as a treatment alternative in LS and other sclerotic collagenoses in both dermatology and rheumatology.

As far as the extensive accumulation of collagen is concerned several investigations demonstrated the long-wave UVA-induced stimulation of the synthesis of specific mRNA-levels of various matrix-metalloproteinases in cultured human fibroblasts, probably due to their lower antioxidant capacity and involvement of the protein kinase C pathway [21,102-110]. Simultaneously, an elevation of interstitial collagenase m-RNA and protein expression can be determined immunohistochemically and by the use of nucleic-acid in-situ hybridization in dermal fibroblasts [21,111]. Furthermore, several studies provided evidence that at least UVA1 irradiation induces the formation of several cytokines and soluble factors e.g. interleukin-1 and/or interleukin-6 stimulating the synthesis of collagenase, while some immunomodulatory cytokines remain unaltered [112,113].

On the other hand, UVA1 irradiation has been shown to initiate apoptotic cell death in dermal T lymphocytes [114,115]. A shift of the balance between protooncogenes (e.g., bcl-2) and tumor suppressor genes (e.g., p53) towards the induction of apoptosis seems to be one of the major effects of UVA1 irradiation [10]. Beside the involvement of singlet oxygen as an early intermediate in collagenase induction, oxidative stress has also been proven to induce lipid peroxidation in cytoplasmatic membranes and to be responsible for DNA damage [116,117]. Therefore, one causal factor of membrane alterations might be the (P)UVA-induced generation of reactive oxygen species, such as singlet oxygens or superoxide anions and hydroxyl radicals leading to lipid peroxidation, structural and functional modifications of membranes characterized by altered fluidity, increased permeability and inactivation of cellular enzymes and transport proteins [116-119]. Furthermore, singlet oxygen is able to open mitochondrial megachannels, releasing apoptosis initiating factor (AIF) and cytochrom c heading towards indirect DNA damage in T cells [116,120,121]. Besides, UVA1-induced apoptosis is triggered by receptor mechanisms, e.g. by the alternative activation of the FAS/FAS-ligand (APO-1, CD95) system in peripheral T cells [114,116,122].

If and to what extent additional modulations of impaired endothelial cells might also contribute to the posttherapeutic clinical and histological improvement still has to be investigated [123]. Nevertheless, the efficacy of different regimens of UVA phototherapy might probably, at least in parts, be due to the mechanisms of action as mentioned above.

Inasmuch as LE and other autoimmune disorders are concerned, a transparent mechanism of action remains obscure. Immunohistologically, LE is at least characterized by an inflammatory T cell derived infiltration mainly of the T helper subtype combined with an impairment of T and B lymphocyte regulation, dysregulated dendritic cell abnormalities and defective clearance of immune complexes and autoantigens [124-126]. Analogous to the sclerosing disorders as mentioned above, induction of apoptosis in T lymphocytes may also represent the committed step of UVA exposure in LE. Simultaneously, UVA has been shown to directly affect presence, function and morphology of dermal and epidermal Langerhans cells, which may result in a suppression of cell-mediated immunity and a disruption of autoreactive T lymphocyte, B cell and Langerhans cell stimulating processes [127-131]. Controversially, UV radiation is often associated with exacerbating skin eruptions and photosensitivity is actually a diagnostic criterion of LE. Nevertheless, in how far improvement of skin condition after repeated irradiation might be due to adaptive decreased oxidative stress upon subsequent UVA exposures has to be evaluated [131].

Despite an extensive therapeutic administration of UVA irradiation, relatively little data are available concerning possible acute and long-term side effects. Usually, patients exhibit a dose-dependent tanning of the skin, which has been described to appear following a single minimal pigmenting dose of 50 J/cm^2 ^UVA1 [132,133], whereas UVA1-induced erythema can usually only be observed after ≥ 90 J/cm^2 ^single dose UVA1 [134]. After UVA1 exposure, individuals of all skin types appear to develop more or less immediate pigment darkening that is due to a reversible photochemical reaction (oxidation of melanin and its precursors and metabolites). In contrast, even after a comparably lower dosage, broad-band UVA irradiation is able to provoke erythema and delayed tanning which is due to an enzymatically controlled production of melanin polymers. After topical or systemic PUVA treatment patients exhibit the characteristic methoxsalen dose-dependent PUVA erythema ranging from three to six days following irradiation [135,136]. Additionally, systemic PUVA is often associated with nausea or vomitus. However, by considering exclusion criteria such as an autoimmune disease associated abnormally increased photosensibility, solar and heat induced urticaria or a history of polymorphous light eruption and, especially in the case of PUVA therapy, by the consequent posttherapeutic use of potent sun protection, additional clinically relevant acute side effects may usually not be expected.

As far as long-term side effects are concerned no definite prediction has yet been taken. Controlled studies dealing with the carcinogenesis induced by broad-band UVA sources are still rare. Nevertheless, the induction of dermal hyperplastic elastic fibers resulting in early skin ageing following a cumulative dose of 4000–8000 J/cm^2 ^UVA1 seems to be imminent [6]. Additionally, as already reported above, the induction of collagenases released by dermal fibroblasts is known to be an important cofactor within this process [9,137]. On the other hand, carcinogenesis of UVA1 irradiation is still poorly understood. Taking the elimination of the potentially procarcinogenetic wavelength ranging from 315–320 nm into account, one might speculate on the possibly lower risk of UVA irradiation [138]. Indeed, possible melanogenetic long-term effects of long-wavelength UV irradiation (induction of malignant melanomas) have previously been discussed [139,140]. Simultaneously, animal studies suggested the induction of squamous cell carcinomas even though provoked by 220 kJ/m^2 ^for a period of 265 days [6]. Until today, no assignment to humans could be achieved. However, at least concentrated PUVA photochemotherapy has been found to be related to potential mutagenesis and the increased occurrence of squamous cell carcinomas as well as malignant melanomas in psoriatic patients [9,141-143].

Moreover, recent investigations concerning the effects of UVA1 irradiation on human dermal endothelium revealed the initiation of apoptotic cascades even after a comparably low dose of single 80 J/cm^2 ^UVA1 radiation. Due to the penetration of up to 20% of UVA to the level of dermal vasculature, the induction of the programmed cell death cascades may develop to one of the main side effects of UVA phototherapeutic strategies [144].

Even though especially in case of LE sunlight exposure has been postulated to induce exacerbation in as much as half of the patients, courses of UVA irradiation can also be used in such disease. In this respect, UVA2 and UVB seem to be responsible for the induction of LE eruptions [145]. Nevertheless, different studies provide strong evidence that apoptosis associated with a shift of the balance between p53 and bcl-2, simultaneously one of the main mechanisms of action concerning UVA phototherapy, may play a role in the pathogenesis and activity of LE and might to correlate with the sequential progress of LE skin lesions [146,147].

As the peak of dermatological therapeutic usage of UVA irradiation still seems to rise, scientific research engagement is needed in order to rate its potential long-lasting negative impact. Therefore, until the evaluation of firm data UVA phototherapy should most likely be restricted in the number of cycles per year, treatment should be supervised by an experienced dermatologist and a UV pass book should be issued.

## Conclusion

Today, by considering this widespread range of clinical and experimental studies, one might clearly conclude that different regimens of UVA phototherapy have simultaneously been developed to effective, often well-tolerated and beneficial therapeutic strategies in the treatment of a variety of sclerotic skin diseases such as SSc, LS, chronic GvHD, extragenital LSA or sclerodermoid rarities and other disorders affecting the connective tissue. In this respect, the controlled application of UVA irradiation seems to exhibit a comparably tolerable risk/benefit ratio as a minimum in case of these precise indications. Furthermore, UVA phototherapy might also be considered as an optional treatment in both the cutaneous and systemic forms of LE, although the mechanism of action remains difficult to understand.

Nevertheless, therapeutic application of UVA phototherapy, especially of long-wave UVA1 phototherapy, is still 'under construction' as to its limited availability apart from selective centers of excellence and mostly uncontrolled pilot investigations or case reports especially as far as PUVA or conventional UVA1 phototherapy in sclerodermic skin affections or autoimmune disorders are concerned. From our point of view there is no doubt that UVA phototherapy could by far be much more frequently used in the treatment of connective tissue lesions of the skin and, due to its ability to affect dermal vascular structure, even of extracutaneous manifestations. However, additional research efforts are required to determine an exemplary clear-cut catalogue of indications responding to UVA irradiation. Therefore, oncoming controlled randomized studies evaluating the efficacy of UVA phototherapy in connective tissue diseases should not only focus on the assessment of further innovative indications, but also on the comparison between phototherapeutic agents and conventional immunosupressive/-modulating regimens as for example systemic glucocorticoids, azathioprine, methotrexate or cyclophosphamide as well as on the confirmation of former uncontrolled reports, not only because of the fact that the natural history of fibrotic disorders includes a period of inflammation/rapid induration followed by a prolonged period of regression even in untreated patients, but also in order to expand its usage to a widely available treatment option.

## Competing interests

None declared.

## Authors' contributions

FB conceived of this investigation including its methods and manuscript structure, performed the comprehensive literature search including data extraction and interpretation, and finished the paper. TG participated in the literature search. A.K. conceived of the study. P.A. participated in its design and coordination.

All authors read and approved the final manuscript.

**Figure 1:**
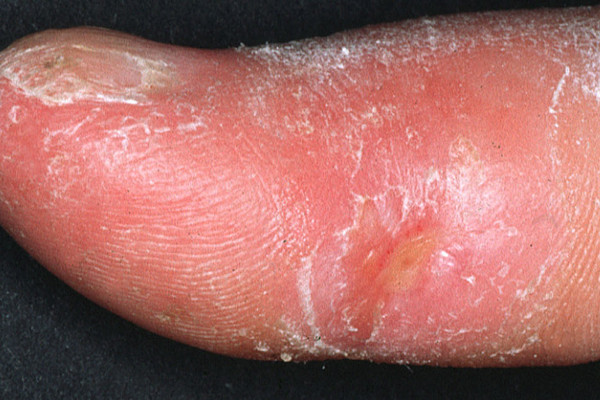
UVA1 phototherapy in systemic sclerosis. Clinical appearence of acrosclerotic piece-meal necrosis of the first digit in SSc before (Fig. 1) and almost complete clearance following low-dose UVA1 phototherapy (Fig. 2).

**Figure 2:**
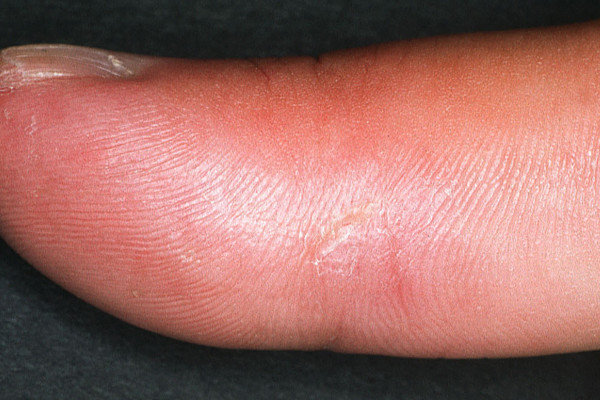
UVA1 phototherapy in systemic sclerosis. Clinical appearence of acrosclerotic piece-meal necrosis of the first digit in SSc before (Fig. 1) and almost complete clearance following low-dose UVA1 phototherapy (Fig. 2).

**Figure 3:**
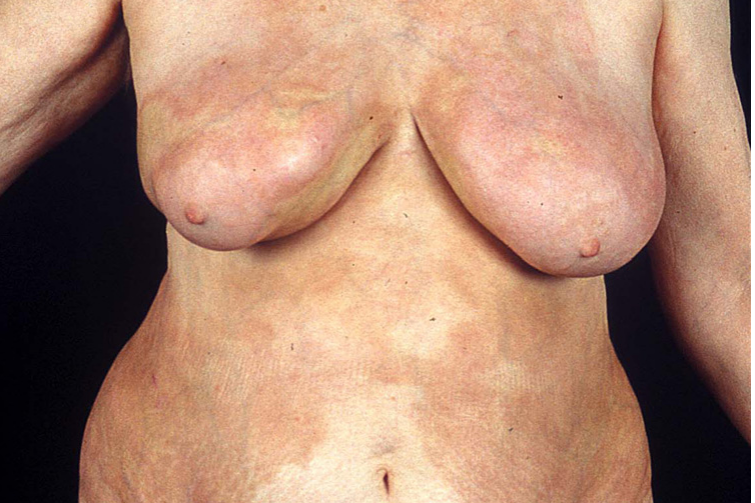
UVA1 phototherapy in localized scleroderma. Macroscopic aspects of LS displaying extensive sclerosis on the chest before (Fig. 3) and after low-dose UVA1 irradiation resulting in a remarkable softening (Fig. 4).

**Figure 4:**
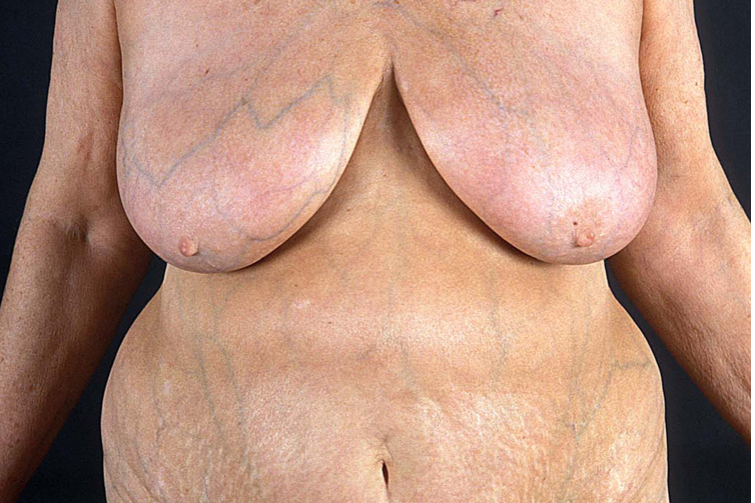
UVA1 phototherapy in localized scleroderma. Macroscopic aspects of LS displaying extensive sclerosis on the chest before (Fig. 3) and after low-dose UVA1 irradiation resulting in a remarkable softening (Fig. 4).

**Figure 5:**
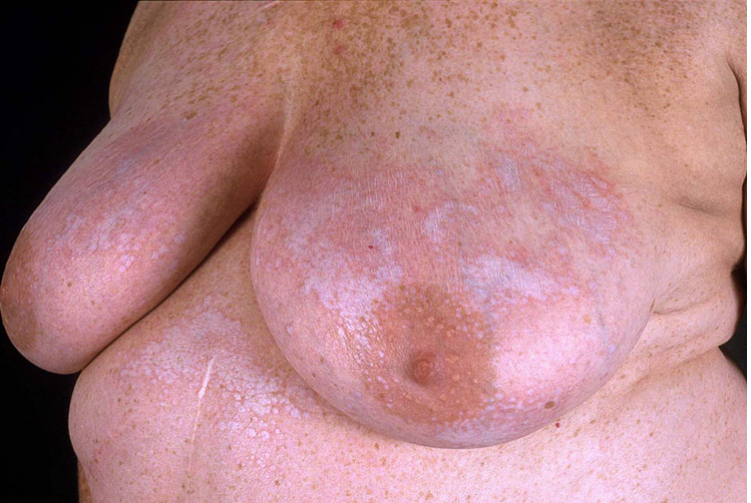
UVA1 phototherapy in extragenital lichen sclerosus et atrophicus. Confetti-like lesions of extragenital LSA (Fig. 5) and marked improvement of following low-dose UVA1 phototherapy (Fig. 6).

**Figure 6:**
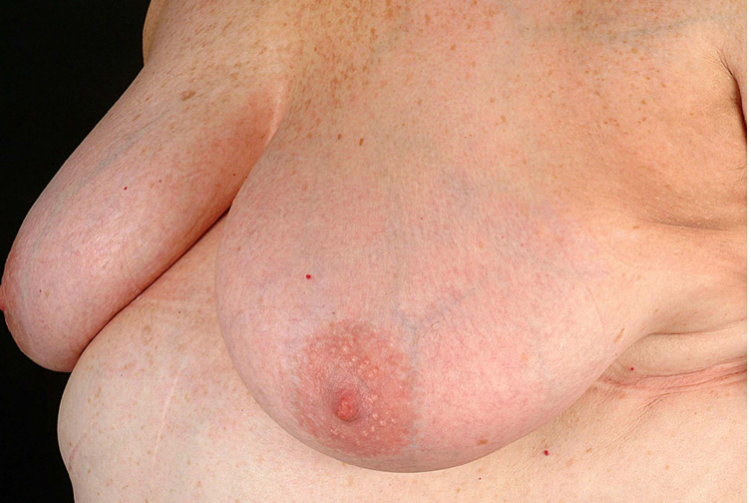
UVA1 phototherapy in extragenital lichen sclerosus et atrophicus. Confetti-like lesions of extragenital LSA (Fig. 5) and marked improvement of following low-dose UVA1 phototherapy (Fig. 6).

**Table 1:** Overview of the different phototherapeutic strategies within the main groups of sclerotic connective tissue diseases. [Categories: A – double-blind, randomized, placebo-controlled; B – open, randomised; C – open, non-randomized; D – case series; E – case report]

**Disease**	**Therapy**	**Dosage**	**Experience**	**Comments**
*Systemic sclerosis*	UVA	-	-	requires evaluation
	UVA1^28–31^	low-/medium-dose	E, C	benefit, especially suited for acrosclerosis and partial body exposure
	PUVA^18,32–34^	medium-dose	E, D	bath application in childhood discussed
*Localized scleroderma*	UVA^36,37^	low-dose	C	benefit, no further evaluation
	UVA1^38–45^	low-/medium-/high-dose	D, C	no exact recommendation in favor to best dosage, benefit, combination with calcipotriol where appropriate, successful in childhood/adolescence
	PUVA^19,32,34,46–51^	high-dose	E, D	questionable efficacy, extreme variance in dosage, combination (cream) with calcipotriol in childhood
*Extragenital lichen sclerosus et atrophicus*	UVA	-	-	requires evaluation
	UVA1^40,56–58^	low-dose	E, D, C	effectiveness, disputable in combined morphea/lichen sclerosis et atrophicus
	PUVA^59,60^	low-dose	E	benefit, careful cream therapy for genitoanal lesions where appropriate
*Sclerodermoid graft- vs-host disease*	UVA	-	-	requires evaluation
	UVA1^62–64^	low-/medium-dose	E, D	partial efficacy, medium-dose possibly more effective than low-dose, combined UV/immunosuppressive therapy
	PUVA^65–71^	medium-dose	E, D, C	skeptical effectiveness, potentially adjunct therapy in addition to conventional chemotherapy, more effective in lichenoid than sclerodermoid lesions
*Lupus erythematosus*	UVA	-	-	requires evaluation
	UVA1^73–78^	low-dose	D, C, A	benefit in occasional cases, long-term application
	PUVA	-	-	requires evaluation

**Table 2:** Synopsis of recent case reports decribing various phototherapeutic alternatives in a number of sclerotic rarities. [Categories: A – double-blind, randomized, placebo-controlled; B – open, randomised; C – open, non-randomized; D – case series; E – case report]

**Disease**	**Therapy**	**Dosage**	**Experience**	**Comments**
*Eosinophilic fasciitis*	UVA1^80^	low-dose	E	benefit, no valid data available
	PUVA^81^	medium-dose	E	
*Pansclerotic morphea*	UVA^84^	low-dose	E	possible efficacy as an adjunct therapy,
	UVA1^83^	low-dose	E	no valid data available
	PUVA^85,86^	medium-dose	E	
*Scleromyxedema*	PUVA^90–92^	high-dose	E	controversial, possible efficacy as an adjunct therapy, no valid data available
*Scleredema adultorum Buschke*	PUVA^95,96^	high-dose	E, D	possible therapeutic alternative, cream therapy, no valid data available
*POEMS*	UVA1^98^	low-dose	E	benefit, no valid data available
*Bleomycin-induced scleroderma*	UVA1^99^	low-dose	E	limited success, no valid data available
*Pansclerotic porphyria cutanea tarda*	UVA1^101^	medium-dose	E	benefit, no valid data available

## Pre-publication history

The pre-publication history for this paper can be accessed here:


